# The Biophysical Effects of Neolithic Island Colonization: General Dynamics and Sociocultural Implications

**DOI:** 10.1007/s10745-017-9939-9

**Published:** 2017-10-25

**Authors:** Thomas P. Leppard

**Affiliations:** McDonald Institute for Archaeological Research, University of Cambridge, Downing Street, Cambridge, CB2 3ER UK

**Keywords:** Ecodynamics, Islands, Neolithic, Convergence

## Abstract

Does anthropogenic environmental change constrain long-term sociopolitical outcomes? It is clear that human colonization of islands radically alters their biological and physical systems. Despite considerable contextual variability in local specificities of this alteration, I argue that these processes are to some extent regular, predictable, and have socio-political implications. Reviewing the data for post-colonization ecodynamics, I show that Neolithic colonization of previously insulated habitats drives biotic homogenization. I argue that we should expect such homogenization to promote regular types of change in biophysical systems, types of change that can be described in sum as environmentally convergent. Such convergence should have significant implications for human social organization over the long term, and general dynamics of this sort are relevant in the context of understanding remarkably similar social evolutionary trajectories towards wealth-inequality not only islands, but also more generally.

## Introduction: Long-Term Social Consequences of Environmental Change?

Are there ineluctable, long-term sociopolitical consequences of anthropogenic environmental change? To what extent are these consequences constrained by—and emergent from—initial socioecological dynamics?. In this paper I address the relationship between radical environmental reorganization and its sociopolitical implications through the lens of human-island ecodynamics. I argue that the anthropogenic introduction of invasive species and the eradication of endemics on islands during the Holocene reduced sum biodiversity in predictable ways that, in theory, should have exercised pronounced homogenizing effects on the function of biophysical systems. Such homogenization should, in turn, have promoted parallel adaptive strategies in discrete human populations.

The arrival of humans on islands throughout the later Quaternary exacerbated extirpation and extinction in endemic biotas already predisposed towards fragility, differentially reducing local and sum global taxonomic diversity. Repeated extinction events wrecked endemic ecologies while intact anthropic ecosystems of domesticates, commensals, and parasites were simultaneously introduced wholesale. In the first section of this paper, I review the evidence for these processes at a global scale, showing that food-producing human populations in particular radically reorganize insular biotas. Moving beyond area-specific studies of insular ecology, I argue that these twin processes of extinction and introduction should have driven ecosystems separated in time and space towards parallel types of organization, a process of biotic homogenization (McKinney and Lockwood 1999). Because abiotic systems (i.e., soil composition; hydrological dynamics) interface with biota to form biophysical systems, emerging anthropogenic homogeneity in biotic organization also drove homogeneity in insular biophysical organization. I utilize modern and experimentally derived data to explore in detail the likely aspects of this biophysical homogeneity, including the emergence of parallel pedological and hydrological dynamics with ramifications for ecosystem organization and function. I build on the general dynamic theory outlined by Whittaker and colleagues (Whittaker *et al.* 2007, 2008; Borregaard *et al.* 2016) and suggest that these processes in sum are best described as environmental convergence, notwithstanding the recognition that islands with varied geological histories may have moved towards convergence along discrete pathways.

My main objective, in linking these well-understood but infrequently synthesized types of process, is to emphasize that discrete instances of prehistoric human colonization of islands should nonetheless in theory drive structurally similar environmental dynamics. Biophysical processes of this sort have sociocultural implications, however. In conclusion, I suggest that these general dynamics may have imposed parallel types of constraint on the development of otherwise unrelated island societies, forcing mitigation strategies centered around capital investment that, in turn, promoted exaggerated wealth inequality. The relationship between environmental constraint, returns on capital investment, and the emergence of sociocultural complexity on islands (as exemplary, on reduced spatial scales, of likely larger and longer-term general dynamics) demands substantive future attention.

This argument is not data-driven, nor does it lean heavily on exemplification via detailed case studies (indeed, in many cases, it is not clear that the data required to support an argument of this sort exist). Rather, it is explicitly deductive. My interest here is in suggesting: (a) that a particular recurring confluence of ontogenetic conditions—the collision of certain types of biophysical organization (insularity) with human food-production subsistence and its associated ecodynamics (introduction/extinction)—necessarily and radically constrains resulting socioecological process; and (b) that the existence of this constraint is simultaneously overlooked but yet extremely significant for social scientists seeking to account for apparent parallelism or convergence in long-term social and political trends, especially so in the current context of large-scale environmental change. The aim is not to propose a series of deterministic causations, but to emphasize that, just as human subsistence and behavioral choices narrow the window of possible environmental trajectories, so this narrowing in turn re-imposes restrictions on the panorama of human behavior.

## Human Colonization and its Biological Effects

### Extinction and Extirpation

The expansion of our genus around the planet has been conditioned by environmental organization, but it has also recursively altered this organization both directly and indirectly. Direct predation, translocation and introduction, and domestication have all driven biotic change, but human modification of the planet (from forest clearance to changing the chemical composition of the atmosphere and ocean) has also had impacts on other taxa. This range of processes has promoted both extinction and the establishment of invasive taxa beyond their native ranges, thereby radically altering ecological structure but also, in the longer term, affecting genetic variability and remolding evolutionary landscapes.

The most obvious examples of anthropogenic extinctions come from the recent past and the present. A preponderance of evidence suggests that the planet is experiencing a mass extinction event (i.e., loss of >75% of extant species within a restricted timeframe) with the likelihood that this process is primarily anthropogenic (Barnosky *et al.* 2011; Ceballos *et al.* 2015). Despite the majority of these extinctions clustering towards the more recent end of the Holocene, the ‘long tail’ of this process extends back into the deeper Quaternary. I ignore here the controversy surrounding whether the extinction of the Pleistocene continental megafauna was driven by human agency, environmental change, or more complex feedback dynamics (Burney and Flannery 2005; Braje and Erlandson 2013; Cooper *et al.* 2015; Stuart 2015; Villavicencio *et al.* 2015; Bartlett *et al.* 2015). What is less open to debate is the profound effect colonizing humans have had on smaller, previously insulated landmasses.

The physiographic organization of islands—as relatively small, discrete types of habitat surrounded by spatially extensive qualitatively divergent habitat—profoundly influences their biotas.^1^ Island biotas (not just faunas, although floras seem in general to respond differently to invasion (Gilbert and Levine 2013; Downey and Richardson 2016)) are peculiarly exposed to extirpation and extinction risks deriving from biological invasions; far more so than continental equivalents (Loehle and Eschenbach 2012; Szabo *et al.* 2012; Weigelt *et al.* 2013). This relates to a number of factors that combine to render insular populations fragile. Most obviously, open ocean impinges on dispersal via filtration effects, reducing gene flow between colonists and source populations and correspondingly driving allopatric speciation. Consequently tending to be rich in endemics, island biotas in total thereby contribute disproportionately to sum global biodiversity, with the result that islands offer more candidate taxa for extinction per unit area than non-insular environments. This threat is exacerbated by the restrictions that insular size imposes on population size (Brose *et al.* 2004) of a given taxon (endemic or not)—keeping populations low and accordingly more greatly exposed to demographic stochastic perturbations (Lande 1993)—and the effective fragmentation ocean promotes in metapopulations (see Rybicki and Hanski 2013). This, alongside the tendency for insular taxa to be very specialized and, in the absence of complex trophic structures, predator-naïve, renders island biotas highly responsive (Fordham and Brook 2010), although clearly and accordingly the degree of insular ecological sensitivity varies along a number of dimensions (including size and remoteness but also geologic antiquity and composition and ‘type’—oceanic (high/atoll), continental) that causes island ecodynamics to vary broadly.

It should be emphasized that invading species—whether humans or their co-traveler taxa, such as commensals or domesticates (Chapuis *et al.* 1994; Nogales *et al.* 2004; Wanless *et al.* 2007)—do not simply exert pressure via direct predation, although clearly this is significant. In affecting the demographic robustness of a species by preying on it (deliberate forest clearance can be understood as a process comparable to predation (McWethy *et al.* 2010)), an invader promotes dynamism in the trophic neighbors of that species, potentially driving ecological release in those it consumes and demographic crashes in those that consume it (O’Dowd *et al.* 2003). The potential for such dynamics to affect the wider system are clear. Importantly, extinction need not be necessary to cause such ecological cascades so much as substantial population reduction, although the permanent removal of a species from an ecosystem (i.e., taxonomic diversity loss) should have observable consequences (i.e., functional diversity loss) (cf. Baiser and Lockwood 2011). Pressure is also exerted by invasive species on ecological relationships beyond the purely oppositional (i.e., predation and competition), with mutualisms, commensalisms, and parasitisms likely to be subject to disruption following extirpation or extinction events (Traveset and Richardson 2006; Sekercioglu 2011; Boyer and Jetz 2014). In general, the processes leading to extinction and extirpation following invasion are complex (e.g., Hanna and Cardillo 2014), but the result is a uniform, gross trend away from elevated biodiversity.

We should accordingly expect the arrival of humans on islands to be transformative. This is borne out in the data. The effect of human colonization (along with co-traveler taxa) on islands is evident from the Upper Palaeolithic, although there exists a possibility that we glimpse ephemeral traces of comparable processes in deep time, with the extinction of the proboscid *Stegodon sondaari* on Flores at 0.9 mya (van den Bergh *et al.* 2009) intriguingly close to the earliest dates for hominin incursion into the Lesser Sundas at 1.02 mya (Brumm *et al.* 2010). The intentional colonization of island groups by hunter-gatherers belonging to our own species, certainly in the Mediterranean, but also in the Caribbean (Steadman *et al.* 2005), tracks closely with the disappearance of suites of endemic species, with 88.9% of mammalian endemics lost in the insular Mediterranean at the Pleistocene-Holocene boundary (Alcover *et al.* 1998). The impacts of hunter-gatherers in Near Oceania are harder to gauge. Steadman *et al.* (1999) suggest anthropogenic avifaunal loss in the Solomons and extinction and range-contraction of the varanids (giant monitor lizards) might be associated with human activity (Hocknull *et al.* 2009). In general, the patchy data hint at a role for humans in the eradication of several taxa including *Stegodon* and the elephantid *Palaeloxodon*, a role which—not least because of sea level rise driving processes of range fragmentation in Sunda and Sahul—is hard to disentangle from broader environmental processes (Louys *et al.* 2007). The same is true of the Californian Channel Islands; extinctions at colonization seem to have been limited to the duck *Chendytes lawi*, but there is evidence for extensive resource depletion and concomitant ecosystem effects (Rick *et al.* 2012; Braje *et al.* 2017b).

A more reliable signature corresponds with the spread of agricultural, agropastoral, or horticultural (i.e., food-producing; ‘Neolithic’ hereafter)^2^ lifeways (see Braje *et al.* 2017a). In the Caribbean and Mediterranean, with their previous exposure to hunter-gatherers, extinctions continued to occur during the establishment and expansion of agropastoral lifeways (e.g., Steadman and Franklin 2015; Bover *et al.* 2016). Remote Oceania, which in contrast had not experienced human presence until the arrival of food-producing communities and was by virtue of its geographic organization rich in endemic taxa, underwent radical change at and beyond human colonization horizons, with endemic avifauna and flora in particular witnessing catastrophic losses (Steadman and Martin 2003; Boyer 2010). These losses are almost certainly in part attributable to large-scale environmental change induced, not only by humans behaviors, but also by ecological release in commensal species, in this case the Polynesian Rat *Rattus exulans* (Hunt 2007; Athens 2009). The Pacific example is striking, not least in the extent to which exaggerated environmental disruption witnessed between 3000 and 500 bp in Remote Oceania prefigures the final cataclysmic arrival of European colonists, their livestock, and their diseases after 500 bp (a topic which I do not consider in detail here). This should not obscure, however, that the rapid traumatization of the endemic Pacific echoed more drawn-out but equally dramatic Holocene eradications in the Mediterranean and the Caribbean.

### Translocation and Invasion

The arrival of humans and their co-traveler species in habitats previously insulated from them drives radical and highly variable ecological change, but the outcome is almost always the same: pronounced biodiversity loss. Such loss is not only an outcome of processes of attrition, however, but also of processes of translocation and invasion. It should be stressed that ecologists distinguish between newly-arrived species according to their long-term reproductive success and degree of associated ecological impact, in terms of *translocated* (taxa that reach a new habitat), *introduced* (taxa that reach a new habitat yet have minimal impact), and *invasive* (taxa that reach a new habitat and have large-scale impact). Many of the taxa discussed subsequently are commonly viewed as invasive (e.g., *Rattus exulans*), yet it is worth emphasizing that it may be the case that many introduced taxa—whose impact is not readily observable at the scales or timeframes at which ecological analysis of invasion tends to be undertaken (e.g., *Thiara* spp.)—may contribute to biodiversity reduction and homogenizing processes. During incipient domestication events around the planet, such species have been bundled together in recurring packages which, in effect, represent artificial ecosystems comprising finite sets of repeated ecosystemic interactions (Çilingiroĝlu 2005). During island colonization, these bundles have often been transplanted wholesale (Kirch 1984), preserving in part extant ecosystemic links between groups of species that co-evolved under the peculiar but aggressively selective conditions of domestication (Boivin *et al.* 2016).

In the Mediterranean, and subsequently much of the planet after 500 bp, translocations included the classic Southwest Asian Neolithic package of cereals and pulses including barley (*Hordeum* spp.) and wheat (*Triticum* spp.) (Willcox 2013; Arranz-Otaeguia *et al.* 2016) alongside domesticated ungulates (most notably cow, *Bos taurus*; pig, *Sus scrofa domesticus*; sheep, *Ovis aries*; and goat, *Capra hircus*) (Zeder 2008). In the Pacific (and across the Indian Ocean, to Madagascar and the Comoros (Crowther *et al.* 2016)), major translocations include coconut (*Cocos nucifera*), taro (*Colocasia esculenta*), yam (*Dioscorea* spp.), banana (*Musa* spp.), and breadfruit (*Artocarpus altilis*), alongside pig, dog, and the domesticated chicken, *Gallus gallus domesticus*. It should be noted that in the Pacific there exists considerable variability among islands regarding the precise composition of domesticated, introduced biotas (for example, all three domesticated fauna rarely co-occur). Moreover, the precise sources of these species and the specific dynamics of their domestication are considerably more contentious than in the Mediterranean, but in general a Southeast Asian and Island Southeast Asian origin is a common theme (e.g., Denham 2011; Gunn *et al.* 2011; Barker and Richards 2013; Pitt *et al.* 2016), with pigs perhaps deriving from domesticated stock in mainland China, but with potential parallel domestication events elsewhere in Southeast Asia (Larson *et al.* 2010; Bellwood 2011). The Caribbean example diverges slightly because of the absence of domesticated ungulates or cereals (excepting of course maize, *Zea mays*) from the Americas. Continental faunas were translocated, however, possibly to provide naturally corralled stocks of non-domesticated protein; instances include armadillo (*Dasypus* sp.), agouti (*Dasyprocta* sp.), guinea pig (*Cavia* sp.), peccary (Tayassu/Pecari sp.), and opossum (*Didelphis* sp.) (Giovas *et al.* 2011, 2016; also Stahl 2009). The extent of deliberate translocations of plant foods is not immediately clear. Sporadic evidence for other early Antillean translocations includes *Manilkara* spp., as Fitzpatrick (2015) notes in providing a comprehensive overview of flora exploited for nutritional purposes during the Ceramic Age (traditionally understood to have involved much more intensive and deliberate management of domesticates and quasi-domesticates than the preceding Archaic), including common bean (*Phaseolus vulgaris*), sweet potato, (*Ipomoea batatas*), and marunguey (*Zamia* spp.), although it is not clear whether the Antilles were included in the native ranges of these taxa. Maize itself—certainly a translocation—is evidenced in human dental calculus from the island Caribbean from around 2000 bp (Mickleburgh and Pagan-Jimenez 2012). Data from Trinidad may push this introduction back to the Mid Holocene (Pagán-Jiménez *et al.* 2015), although the insular status of the island during Mid Holocene seastands is unclear and, even if Trinidad was insular during the Mid Holocene, the over-water distance to the South American mainland would have been negligible.

Various other taxa have been translocated accidentally, such as the house mouse *Mus musculus domesticus*, as well as various species of shrew, during Mediterranean Neolithicization (Vigne 1988; Cucchi *et al.* 2005), and the aforementioned Polynesian Rat, *Rattus exulans*, during the colonization of Polynesia (although there is debate regarding whether this was indeed an accidental or deliberate translocation (Matisoo-Smith *et al.* 1998; Allen 2015)). Similarly, murids of several types were unintentionally translocated in the Indian Ocean (Fuller *et al.* 2011). The process was not limited to vertebrates; other instances include the translocation of the aquatic snail *Thiara* spp. and other molluscs to Remote Oceania, potentially in the context of wetland taro cultivation (Kirch 1996; Kirch *et al.* 2009). There are also other, less archaeologically conspicuous examples. Movement of exotic pathogens has attracted most attention within the context of European expansion and colonialism after 500 bp (e.g., Nunn and Qian 2010), but colonizing Neolithic humans inevitably translocated novel pathogenic bacteria and viruses (as well as relatively benign unicellular organisms; e.g., gastrointestinal microbiota) to previously insulated environments, provoking new, if now unrecoverable, ecological relationships.

In the Mediterranean, the Caribbean, and the Pacific, Neolithic colonization—essentially comprising a series of repeated biological invasions—drove substantial change in biotic organization. These processes were to an extent regionally specific (as regards the ecological coherence of discrete types of Neolithic package) but, in terms of general process, are comparable; I return to this distinction shortly. These initial Neolithic island colonizations were followed by both intentional and accidental translocations, as well as extinctions (Ratcliffe and Calaby 1958; Helmus *et al.* 2014), on a prodigious scale, associated with the European expansion over the last half millennium. This second phase of introductions has clearly exacerbated and more comprehensively generalized these biotic changes. The time depth of these processes beyond the Late Holocene (and concomitant implications for human social development) is, however, less frequently emphasized, and accordingly I focus on initial Neolithic colonization. Specifically, I suggest that drastic, short-term reduction in sum biodiversity on islands is essentially a process of homogenization. This homogenization is likely to have had effects and these effects in turn should have severely constrained the landscape of human subsistence choices in the medium to long-term.

## Neolithic Colonization as a Driver of Biotic Homogenization

The effect of island colonization by humans over the recent Quaternary has not simply been loss of biodiversity and destruction of endemic ecosystems, but replacement of these ecosystems with highly anthropogenic portmanteau ecologies. Evidently, at local scales each ecological situation is contextually unique, and we should expect different types of insular geologic and biogeographic environments to exhibit varied responsiveness, but the repeated introduction of species with finite behavioral repertoires encouraged similar types of ecosystemic relationships; for example, impressively varied late Pleistocene ungulate herbivory on insular Mediterranean *maquis* flora, involving a range of higher taxa, was replaced during the Holocene largely by two species of Caprini. This is a process of biotic homogenization.

Biotic homogenization occurs when human activity affects the organization of biota in a non-random fashion, negatively affecting a larger number of taxa and positively affecting a smaller number. The outcome, as large numbers of taxa undergo extinction and smaller numbers of taxa experience range expansion, is reduced spatial biodiversity (McKinney and Lockwood 1999; Olden 2006). Taxonomic variety should not be simply equated with behavioral diversity; ecological roles performed by a given endemic species may, following its eradication, have been closely approximated by an invasive species, and we cannot assume that loss of ecosystem function necessarily accompanies species loss (i.e., we must differentiate taxonomic and functional homogenization (Olden *et al.* 2004; Baiser and Lockwood 2011)). In general, however, we can expect repeated eradication of endemics and introductions of very limited biotic suites to suppress behavioral diversity in a manner comparable to, if not matching in extent, loss of genetic diversity, driving the emergence of dominant, repeated sets of ecological interactions.

Different types of environment are likely to witness different rates and types of homogenization process. There is reason to suppose that the same factors that make island biota relatively responsive may also combine to make them more susceptible to homogenization processes (Cassey *et al.* 2007). This perhaps in part relates to the possibility that homogenization is greater at low species richness (Olden and Poff 2003), a condition characteristic of insular environments, and that it depresses those factors that encourage allopatric speciation (Olden *et al.* 2004). While the general trend is towards insular biotic homogenization (Rosenblad and Sax 2016), it does appear that fauna and flora gravitate towards homogeneity at differing rates (Shaw *et al.* 2010; but Kueffer *et al.* 2010), although scales of temporal analysis adopted affect assessment of overall process (Rosenblad and Sax 2016). The key recognition, based on the foregoing review of extinctions and translocations, is that we can establish homogenization as an active ecological process with a substantial time depth, potentially up to and beyond the Pleistocene-Holocene boundary (although more recent in, e.g., Remote Oceania).

I suggested above that these processes of homogenization were regionally specific regarding the ecological coherence of Neolithic packages but, in terms of general process, were comparable. Unlike current homogenizing processes, individual island theaters associated with discrete Neolithic packages experienced greater intra-regional than inter-regional convergence prior to 500 bp; related invasive dynamics on, for example, Mid Holocene Cyprus and Crete drove greater homogeneity between them than between Cyprus and Late Holocene Puerto Rico. This is not substantially problematic, however, as—from the perspective of the wider implications of processes, rather than within a context of conservation biology—it is the outcomes of such processes, rather than the degree of sum global homogeneity, that is most relevant.

This review of data for extinction and translocation indicate that biotic homogenization has been an active ecological process on islands over the later Quaternary. I now explore the possibility that biotic homogenization promoted similar processes beyond the biosphere. In particular, that homogenizing biotas, interfacing with abiotic systems, should have driven regular and repeated biophysical changes that resulted in processes of insular environmental convergence.

## Biophysical Outcomes of Insular Biotic Homogenization

Biota interface with abiotic physical systems. Various organic including growth, metabolism, mobility, and decay involve the structural reorganization of the abiotic environment around the organism. Accordingly, it is appropriate to describe those aspects of environmental systems in which biotic and abiotic processes are especially intertwined (most notably pedology and hydrology) as biophysical systems. Biotic homogenization has implications for insular biophysical systems.

### Soil Biogeochemistry and Integrity

Soils interface with organisms in a variety of qualitatively discrete yet related ways, and are central to ecosystem organization and function (Vereecken *et al.* 2016). Most obviously, microbiota in soils form parts of larger ecosystems, but more strictly biophysical relationships include the manner in which biota affect the biogeochemical composition of soils, as well as their mechanical and structural properties. An important resulting recognition is that structural cohesion of soils is not independent of biogeochemical and taxonomic diversity in soil and associated plant communities, and that the types of biotic homogenizing processes evident on islands during Holocene colonization have, in other contexts, driven predictable processes as regards soil content and integrity.

The composition of plant communities is a primary determinant of soil biogeochemistry, as flora and soils exist in dynamic biochemical feedback relationships (plant-soil feedbacks (PSFs), Ehrenfeld *et al.* 2005). Accordingly, changes in the composition of plant communities during the transition from more heterogeneous to more homogeneous biota have implications for pedological dynamics. Community composition, as noted, can be affected along a variety of dimensions; invasive plants can outcompete and replace endemics, and behavior in faunas—most obviously herbivory, but also including mobility and associated disturbance, excretion, composition of gastrointestinal microbiotas, etc.—homogenizing along similar gradients can drive parallel types of change in soil nutrient organization (e.g., Sánchez-Piñero and Polis 2000). We can deal first with plant-plant interactions. Plant-soil feedbacks are interrupted or modified during biological invasions (Wolfe and Klironomos 2005; Stinson *et al.* 2006), although the manner in which these disruptions occur is not fully understood (Suding *et al.* 2013; Schittko *et al.* 2016). What is clear is the capacity of invasive species to interrupt plant-soil feedbacks to their own comparative advantage and to the disadvantage of native species (Callaway *et al.* 2004; Perkins *et al.* 2016), constructing preferentially less biogeochemically diverse soils. In addition to community turnover in strictly floral terms, herbivory on the part of invasive and comparatively Homogeneous faunas (especially but not only domesticated ungulates) may adversely affect native plants more severely than invasive species in a series of manners, either biased against them because of their relative ecological naïveté or, where there is no clear herbivorous preference for endemics versus exotics, nonetheless having greater effect on endemic species via smaller population sizes and resulting greater exposure to demographic stochasticity. The sum effect of biotic homogenization, via these varied impact pathways, is radical reorganization away from diversity in soil biogeochemistry.

The effects of homogenization in soil biogeochemistry are substantial; not only in terms of of the constraints that homogeneous soils subsequently impose on ecosystem development as regards biomass potential and nutrient availability (in the context of different plant taxa possessing different requirements (Marschner and Marschner 2012)), but also in terms of pedological structural properties. Soil chemistry has implications for the robustness of pedological units insofar as it determines the spatial distribution of community members and thereby root structure, which directly promotes soil integrity (Bergmann *et al.* 2016). In the context of PSF dynamics in which increasing homogeneity in soil biogeochemistry promotes biodiversity-loss in communities (Perkins *et al.* 2016), experimental work that suggests that decreased floral biodiversity correlates positively with inability of soils to resist erosion is consequently significant (Berendse *et al.* 2015); more so if exacerbated soil loss and decreased biodiversity exist in a feedback relationship (cf. Garcia-Fayos and Bochet 2009; Bergmann *et al.* 2016). Bautista *et al.* (2007) find that higher functional diversity corresponds with lower runoff because of the relationship between higher diversity and patch density. These studies indicate that biogeochemical homogenization of soils drives community dynamics that in combination negatively affect soil stability and retention.

It is not only shifts in biogeochemical organization associated with root depletion that promote macro-scale change in soils, and here we can briefly consider ungulate herbivory and its pedological effects in terms of direct mechanical impacts. Comparative data from the relatively recent introduction of the domestic goat *Capra hircus* to the Pacific and South Atlantic suggest that substantial biophysical change might be anticipated during comparable introductions in the Early-Mid Holocene insular Mediterranean. Goat herbivory is implicated in erosion of topsoils (Mwendera *et al*. 1997; Yong-Zhong *et al*. 2005; Yocom (1967) reports 1.9 m of soil lost from the Haleakalā Crater on Maui since introduction). This derives from their eradication of native (as well as invasive) flora and consequent sub-surface biomass loss (Cronk 1989; Chynoweth *et al*. 2013), capacity to create ecological conditions that favor invading taxa (Wilcove *et al*. 1998), and the associated breakdown of nutrient-cycling (Hata *et al*. 2014), processes that are intrinsically interlinked. While the capacity of *Capra* to drive parallel types of biophysical reorganization in invasions is substantial, the domestic pig *Sus scrofa domesticus* was introduced prehistorically not only to the insular Mediterranean but also the Pacific, driving similarly large-scale and parallel biophysical change. The omnivory of pigs can promote predictable ecological dynamics at broad spatial scales. Hawai’ian studies indicate that the most conspicuous effects of pig behavior are reduced growth and survival in plant prey-species (Cole *et al.* 2012; Murphy *et al.* 2014; see also Campbell and Rudge 1984), which has profound implications for soil structure. On O’ahu, areas with pigs present versus areas with pigs excluded experienced much more substantial runoff, associated with the effects of pig-rooting (Dunkell *et al.* 2011). Nogueira-Filho *et al.* (2009) note that effects associated with the presence of pigs also include facilitation of dispersal of exotic flora and soil degradation via trampling and mobility. Overall, in contexts where biotic homogenization includes as a main component the introduction of stocks of domesticated mammals, there is an evident cross-cultural regularity in resulting pedological processes.

Other taxa beyond introduced ungulates drive homogenizing processes in the biogeochemistry and structural dynamics of island soils. Studies of the emergent environmental properties of commensal invasions focus to some extent on human-mediated introductions to very biogeographically isolated islands after 500 bp, but we can in part retroject these ecodynamic trajectories onto Early-Mid Holocene colonization events. Impacts of commensals, especially murids, have attracted most attention (Angel *et al.* 2009; Bolton *et al.* 2014; Simberloff 2009), not least in their capacity to adversely affect nutrient input (especially of phosphorous and potassium) via predation on seabirds, which otherwise act as biogeochemical vectors between marine and terrestrial ecosystems (Mulder *et al.* 2011), although other modes of predation also drive down taxonomic biodiversity. The general trend is again one of impoverishment, with human commensal species contributing to processes of biogeochemical soil depletion—and consequently sum homogenization—alongside deliberately introduced fauna.

What are the main implications of insular biotic homogenization for pedological structure? Biotic homogenization is equivalent to loss of variability, and this is mirrored in biophysical systems. A direct consequence of the agropastoral colonization of islands should be increasingly parallel dynamic soil processes: loss of heterogeneity in the type of PSFs encouraging expansion of exotics at the expense of native taxa, with resulting impoverished and uniform patterns of nutrient distribution reducing below-surface biomass and degrading pedological integrity. This in turn promotes predictable changes in soil dynamics at large spatial scales, as the hydrological system interacts with soils. Root biomass is a primary inhibitor of rill erosion (Gyssels *et al.* 2005); decreasing root biomass diminishes the efficacy of vegetation as a break on the evolutionary transition from splash/sheet erosion to rill/gully erosion (Woodward 1999; Di Stefano *et al.* 2013) (a function also of slope gradient and rainfall dynamics), with the latter two types accounting for >90% of actual sediment transport (Fang *et al.* 2014).

### Erosion, Hydrology, Sedimentation

The movement of large amounts of sediment around the landscape by water has ramifications along a number of axes. It substantially reconfigures the distribution of soil biogeochemical components (whether derived from biotic processes or from weathering of geological substrate), in gross terms moving them downslope and towards—and sometimes beyond—the coastline (Pimentel and Kounang 1998; Pimentel 2006). This redistribution involves both the deposition of the material elsewhere, and sum material loss (Ritchie *et al.* 2007). For example, studies of available soil organic carbon (SOC) have found that SOC eroding from upland soils is differentially retained in downslope soil formations (Nadeu *et al.* 2014; cf. Kirkels *et al.* 2014), while some carbon is lost through fluvial transport to, ultimately, the sea. Similar dynamics apply to nutrients, in particular nitrogen and phosphorous; complex interactions between these various components notwithstanding, disruption of nutrient-cycling and general degradation of soil quality is evident generally, especially at parent soils (Quinton *et al.* 2010). We are essentially dealing with two types of degradation: absolute nutrient loss from the environment, and spatially specific reorganization of the nutrient landscape. The major outcome of this is overall impoverishment of the pedological environment, but a secondary outcome is preferential distribution to areas of deposition and deleterious effects in eroding areas. As nutrient distribution is an important determinant of autotroph biomass (Marschner and Marschner 2012), sediment transport forecloses on the sustainability of thresholds of growth in some areas and provides a new basis for growth in others. It should be emphasized that there is a temporal lag in processes of redistribution that is important at timescales relevant for biota and ecosystem function; soil destruction and movement (i.e., transition to sediment) can be rapid, but pedogenic processes after sediment deposition are much less rapid (Vereecken *et al.* 2016). The recursive consequences of soil dynamism for ecosystem organization are substantial.

Sediment movement via water transport changes landscape morphology (notably slope), which recursively alters hydrological dynamics, driving up flow-rates in areas of higher relief and retarding waterflow across plains as sediment is deposited. Hydrological organization is a dynamic system, with this dynamism constrained non-randomly by various factors including slope and sediment input and transport (Perron *et al.* 2012; Willett *et al.* 2014). Accordingly, increased sediment input into river systems via increased erosion alters the extent to which this input exercises constraint on hydrological dynamics. This has biological implications, as well as those for the physiographic organization of landscapes; increasing volumes of water-borne sediment have impacts on riverine and lacustrine ecosystems, affecting trophic webs and reproduction (Wood and Armitage 1997). Beyond terrestrial hydrological systems, studies suggest that terrigenous sediments can radically disrupt near-shore (and especially lagoonal) environments (Fabricius 2005). We cannot directly correlate modern with prehistoric sedimentation as the chemical burden of modern suspended sediments is likely to be more diverse and more deleterious than in Early-Mid Holocene near-shore deposition. Nonetheless, evidence that modern sedimentation can reconfigure coral reef ecosystems via a series of impact pathways beyond those related to toxin accumulation is significant; suspended particles drive changes as diverse as inhibiting photosynthesis by obstructing photic penetration of the water-column (Storlazzi *et al.* 2015) to altering feeding behavior in fish, as well as entering the trophic system via ingestion (Tebbett *et al.* 2017). Sedimentation associated with interior island erosion following episodes of prehistoric colonization should be expected to have influenced marine ecosystems along similar pathways, with this influence not only limited to tropical latitudes (e.g., Airoldi and Cinelli 1997).

Downslope sediment transport denudes uplands, exposing geology that had been previously covered by regolith. Weathering of such geology is considered to be central to several biophysical processes, not least the carbon cycle (e.g., Maher and Chamberlain 2014), leading to the introduction of new chemicals to the zone in which the lithosphere and atmosphere interact (Anderson 2012). The potential clearly exists for nutrient losses associated with erosion to be replenished by fresh weathering, although (a) with altered structural conditions in the regolith the release and distribution of such nutrients is likely to be very variable, and (b) heterogeneity in the underlying geology may mean that types and proportions of nutrients released do not conform to previous nutrient ratios. Plant-soil feedbacks suggest that any establishing plant community will, other factors being equal, accordingly diverge from the structural composition of the preceding community.

Clearly, having stressed the degree to which biotic and physical systems intermesh in a series of feedback relationships, it would be possible to continue to track the subsequent implications of these (and other) biophysical outcomes of biotic homogenization, but to do so would be to labor the point. It is evident that various types of ecological processes can cause cascade effects that move through biophysical systems. These effects, in reorganizing physiographic structure, then re-frame the physical conditions which constrain biota, and in this sense this relationship is recursive, and likely to obtain in different instances of island colonization.

## The General Dynamics of Neolithic Island Colonization

### Environmental Convergence

Data clearly suggest that agropastoral colonization of previously insulated habitats during the later Quaternary initiated (via a dual mechanism of extinction and translocation) a series of homogenizing biotic processes. Accordingly, these anthropogenic insular biotas came to more closely resemble one another in terms of relative lack of taxonomic and functional diversity. As biota interface with abiotic systems, so increasingly homogeneous biotas in increasingly homogeneous ecosystems interfaced with biophysical systems. Modern and experimentally derived data on biophysical dynamics suggest that this process would have driven repeated types of dynamics across a range of scales, from the very small (soil biogeochemistry) to the very large (alluvial formation), admitting that the rate and severity of these dynamics should vary significantly depending on local ontogenetic conditions.

This suggests that the central dynamics of post-colonization insular environmental change should be predictable (Whittaker *et al.* 2007, 2008; Borregaard *et al.* 2016). Whittaker *et al.’s* ‘general dynamic theory of oceanic island biogeography’ (Whittaker *et al.* 2007; Whittaker *et al.* 2008) adds a further dimension to the original assumptions of MacArthur and Wilson (1967), emphasizing that the physiographic organization of small volcanic oceanic islands changes over evolutionary time in ways that are generally knowable (Borregaard *et al.* 2016). In the case of volcanic islands, the taxonomic diversity of each can be contextualized within its position on the temporal spectrum from initial formation through the cessation of volcanic mass-building to erosion and ultimately atoll formation. The evolutionary specifics of the model are not here of immediate interest; more relevant is the notion that initial ontogenetic conditions, interacting with biota, constrain resulting biophysical processes within certain parameters. This concept can be utilized to consider interactions between human colonists and insulated environments in terms of a general dynamic theory. Despite evident diversity in the cultural component of such colonizations, the ecodynamic outcomes are largely similar: Neolithic colonization and its predictable biotic effects impose constraints on biophysical systems such that their organization on islands comes to more closely approximate other such systems. The general outline—parallel types of contextual constraint driving unrelated morphologies towards a common form—suggests that we may best consider this a process of environmental convergence.

I use convergence here in a loose sense to describe a process in which two entities come to resemble one another in their present present morphologies yet whose resemblance masks deep initial morphological variation. In evolutionary biology, convergence is increasingly seen as having a genetic basis; even at macro scales, it is understood to be an outcome of explicitly Darwinian processes. (Losos 2011; McGhee 2011; Stern 2013). Demonstrably, inorganic systems are not under Darwinian selective pressure. Natural selection itself, however, is one of many structured sorting processes, and this provides a useful insight into considering the nature of insular biophysical convergence. With very different pre-colonization biophysical conditions, the suite of effects associated with colonization by agropastoral humans exercised comparable types of ecodynamic pressure on islands. Because of the structural parallels in how island biophysical systems are organized, these comparable types of pressure drove comparable emergent outcomes: specifically, biotic homogenization followed by abiotic dynamics associated with homogenization and biodiversity loss. These cascades were recursive, with the biophysical outcomes of biodiversity loss driving greater biotic homogenization. Accordingly, the structural organization of island environments separated in time and space converged to more closely approximate one another.

The goal of this paper has been to emphasize that prehistoric human colonization of islands ineluctably forces biophysical changes along limited and consequently quasi-predictable axes. Recognizing that the human capacity to alter biophysical systems such that environmental convergence is the outcome has broader implications, not least for the scholarship regarding the nature and time-depth of the Anthropocene (e.g., Braje and Erlandson 2013). However, I now briefly consider how anthropogenic environmental convergence may have iteratively affected social and political organization of island communities in the aftermath of colonization.

### Sociopolitical Implications: Mitigation, Capital Investment, and Wealth Inequality

From the perspective of human landscape management, the general dynamic landscape processes sketched above are mostly deleterious, in particular soil and nutrient redistribution, but also degradation of marine environments and unpredictability in hydrological organization. Clearly, deleterious processes of this sort (whether anthropogenic or not) have occurred across the planet, but the foregoing discussion suggests we might expect them to be more rapidly or acutely experienced on islands. It is a reasonable assumption that Neolithic societies cross-culturally and characteristically act to mitigate processes viewed as deleterious, in general acting to boost resilience and minimize risk in the short-medium term (e.g., Quintus *et al.* 2016). I now address how these general environmental dynamics may have prompted strategic responses from Neolithic communities.

Strategies to mitigate such processes would cluster around attempts to limit pedological loss (and boost productivity) via capital investment; programs might take the form of terracing, hydrological management, and nutrient replacement. Diversification within horticultural/agropastoral regimes should also serve to offset calorific losses associated with upslope soil depletion. Recognizing the relatively high productivity of coastal environments in mid- and low-latitude contexts (especially within the latitudinally-determined coral belt), diversification into and increasingly intensive exploitation of offshore resources might also be expected. There is some supporting evidence for this, notably for terracing (e.g., Bevan and Conolly 2011; Quintus *et al.* 2016) and soil-management (e.g., Ladefoged *et al.* 2005) in the Mediterranean and Pacific, as well as fishpond aquaculture in Hawai’i.

Clearly, investment in landscape capital is not an island-specific phenomenon. Nonetheless, in the face of substantial change in biophysical systems deriving from colonization impacts, we might expect such investment in island contexts to be: (a) comparatively more spatially expansive than in non-island contexts, because of biophysical disruption affecting more of the total productive landscape; (b) more costly per capita, because of a relatively low population/high investment cost ratio; and (c) more central to maintaining surplus flows, because capital-intensive systems such as terracing or aquaculture should constitute a greater proportion of the total subsistence system.

Essentially, to mitigate risk, non-hierarchical societies with limited inter-generational transmission of wealth would have initially invested heavily in spatially heterogeneous programs of landscape modification. This would have significantly transformed how quickly and in which parts of the community surplus accumulated, with spatially variable landscape investment driving spatially (and thereby socially) variable patterns of productivity and thereby allowing capital to aggregate rapidly and unevenly. Differential investment of labor and resources in mitigatory infrastructure may be more significant in the context of insular social organization often counter-intuitively exhibiting evidence for hierarchical and complex social forms. This runs against the grain of prevailing social evolutionary theory and its emphasis on demographic blocs and large surpluses.

Clearly, linking landscape capital and emergent systems of inequitable wealth distribution is not an original argument; however, this should be contextualized within our understanding of insular carrying capacity and recent discussions of the precise socioeconomic mechanisms that can drive exaggeratedly skewed wealth-distributions. Piketty (2014), exploring conditions that may promote or depress wealth inequality, demonstrates that very skewed wealth-distributions may emerge in contexts of comparatively high returns on capital and comparatively depressed overall growth. This has a central, hitherto under-appreciated relevance as a mechanism for explaining emergent inequality in low productivity environments. Island contexts—relatively marginal compared to, for example, extensive continental patches of fluvial or loessial Quaternary sediments—should permit relatively low overall socioeconomic growth under conditions of Neolithic subsistence strategies. Investment of landscape capital by those segments of society with the capacity to do so should drive exaggeratedly high returns relative to comparable non-insular (i.e., a more productive and resilient) contexts. This approximates a low growth-high capital returns scenario, driving greater disparities in wealth than in more productive or less biophysically responsive environments. This is beyond the scope of this paper, but addressing the extent to which finite and predictable responses to general ecodynamic processes drive similarly comparable emergent effects in terms of social organization and wealth disparity is an important focus for future research.

## Conclusions

Islands, despite their evident diversity in terms of physiography and biology, share certain ecological tendencies in terms of responsiveness to invasion. Neolithic packages, also evidently enormously diverse, nonetheless are organized such that their introduction tends to radically destabilize island biota. A range of data proxies indicate that this destabilization manifested along several predictable pathways during the Neolithic colonization of Mediterranean, Pacific, and Caribbean islands. The predictable and recognizable effects of biological invasions should, I suggest, be evident also in those physical systems that interface with biota, which experience predictable types of process as they converge via a general ecodynamic trajectory. This process of environmental convergence has significant implications for insular human ecology, not least in terms of providing routes into testing spatio-temporal models of maritime dispersal and highlighting the deep history of human impacts on the geosphere, rather than simply on the biosphere.

Beyond the implications more usually considered within the framework of human or historical ecology, this model has ramifications for social organization itself that I have addressed here only briefly. Environmental convergence would have demanded mitigation strategies on the part of island colonists, in particular comparatively expansive (for small-scale Neolithic societies) programs of capital investment. Island environments nonetheless remain comparatively marginal, and it is in the dynamic tension between capital investment, growth, and returns (different ratios of which can drive or suppress emergent inequalities), Neolithic egalitarian ideologies, and marginal and less resilient environments (islands are exemplary, but perhaps not unique in their biophysical responsiveness) that future work may locate the kernel of emergent insular social complexity.

Finally, I stress that islands are not essentially representative of qualitatively distinct or otherwise unique types of environmental organization. Instead, they are illustrative of broader processes by virtue of the extent to which they are exemplary of the tendency of the surface of the geosphere to be both heterogeneous and scalar in this heterogeneity. In this sense, then, the issue becomes one of relative scale (cf. Brose *et al.* 2004), and we might suppose that the types of socioecological constraint sketched above are noteworthy in insular perspective only to the degree that they are felt more acutely and rapidly in more restricted ‘island’ contexts than others. Recognizing that these dynamics anticipate larger-scale and longer-term continental processes would contribute substantially to bridging the divide between palaeoenvironmental and ecodynamic research on the one hand, and models of social evolutionary development on the other.

## Footnotes

1 Clearly, in this sense, Afro-Eurasia is just as insular as Tristan da Cunha; interest lies in scalar difference covering—in this instance—almost five orders of magnitude permitting trophic systems whose difference in complexity (measured, for example, either in species number or total population size) probably exceeds five orders of magnitude. See Brose *et al.* 2004.

2 ‘Neolithic’ affords such semantic latitude that, depending on how it is defined, it becomes more or less inappropriate contextually. What is necessary, however, is a term which embraces hugely variant situations in which, nonetheless, human communities derived the majority of their terrestrial calories from biota which had gone through, or were going through, processes of domestication. Clearly, the extent to which populations were reliant on terrestrial domesticated biota varied substantially from instance to instance, but nonetheless represents, in all cases, a qualitative break with previous subsistence strategies. Consequently, ‘Neolithic’ is preferred as the least-worse option.
